# Naturally occurring proteinaceous nanoparticles in *Coptidis Rhizoma* extract act as concentration-dependent carriers that facilitate berberine absorption

**DOI:** 10.1038/srep20110

**Published:** 2016-01-29

**Authors:** Bing-Liang Ma, Chun Yin, Bo-Kai Zhang, Yan Dai, Yi-Qun Jia, Yan Yang, Qiao Li, Rong Shi, Tian-Ming Wang, Jia-Sheng Wu, Yuan-Yuan Li, Ge Lin, Yue-Ming Ma

**Affiliations:** 1 Department of Pharmacology, School of Pharmacy, Shanghai University of Traditional Chinese Medicine, Shanghai, China; 2 School of Biomedical Sciences, The Chinese University of Hong Kong, Hong Kong SAR, China; 3 Department of Physics, The Chinese University of Hong Kong, Hong Kong SAR, China; 4 Experiment Center for Science and Technology, Shanghai University of Traditional Chinese Medicine, Shanghai, China

## Abstract

Pharmacological activities of some natural products diminish and even disappear after purification. In this study, we explored the mechanisms underlying the decrease of acute oral toxicity of *Coptidis Rhizoma* extract after purification. The water solubility, *in vitro* absorption, and plasma exposure of berberine (the major active compound) in the *Coptidis Rhizoma* extract were much better than those of pure berberine. Scanning electron microscopy, laser scanning confocal microscopy (LSCM), and dynamic light scattering experiments confirmed that nanoparticles attached to very fine precipitates existed in the aqueous extract solution. The LSCM experiment showed that the precipitates were absorbed with the particles by the mouse intestine. High-speed centrifugation of the extract could not remove the nanoparticles and did not influence plasma exposure or acute oral toxicity. However, after extract dilution, the attached precipitates vanished, although the nanoparticles were preserved, and there were no differences in the acute oral toxicity and plasma exposure between the extract and pure berberine. The nanoparticles were then purified and identified as proteinaceous. Furthermore, they could absorb co-dissolved berberine. Our results indicate that naturally occurring proteinaceous nanoparticles in *Coptidis Rhizoma* extract act as concentration-dependent carriers that facilitate berberine absorption. These findings should inspire related studies in other natural products.

Natural products have long been used in the form of crude extracts to treat diseases in ethno-medical traditions such as traditional Chinese medicine (TCM), Ayurveda, and traditional Arab medicine. Natural products are also rich sources of compounds with novel structures^1^,^2^. Frustratingly, the bioactivities of some natural products diminish or disappear after separation and purification^2^, which may be associated with the loss of pharmacodynamic and/or pharmacokinetic synergies among natural product constituents^3^,^4^. According to the theory of multi-target therapeutics^5^, the pharmacological effects of natural products may not be caused by a single compound, but instead may be a result of the synergistic combination of the constituents^6^,^7^. On the other hand, constituents of natural products may enhance the effects of active compounds by improving their pharmacokinetic properties^8^,^9^,^10^,^11^,^12^,^13^,^14^. However, the detailed mechanisms underlying the synergistic effects of natural product constituents remain to be elucidated for most natural products with therapeutic uses.

*Coptidis Rhizoma*, a widely used TCM, is produced from the dried roots of plants of the family Ranunculaceae, such as *Coptis chinensis* Franch^15^. A high dose of orally administered aqueous extract of *Coptidis Rhizoma* showed acute toxicity in mice and led to acetylcholinesterase inhibition-related death^16^,^17^. The aqueous extract of *Coptidis Rhizoma* also showed dose-dependent hepatic and pulmonary sub-chronic toxicity in Sprague-Dawley rats: a low dose of the extract (1.88 g/kg) did not cause adverse effects, whereas a higher dose (3.76 g/kg) caused significant damage^18^. The mechanism of the severe dose-dependent toxicity was not clear. Clinically, the dose and treatment period of *Coptidis Rhizoma* are strictly restricted to avoid adverse effects^16^.

Several alkaloids (Fig. 1), including coptisine, palmatine, jatrorrhizine, and especially berberine, are the dominant active compounds in *Coptidis Rhizoma*^19^. Berberine has been reported to have multiple pharmacological effects^19^ and was introduced into clinical practice in Europe in 2014 because of its beneficial effects as a treatment for type 2 diabetes mellitus and its capacity to improve lipid metabolism in patients^20^. Orally administered pure berberine showed weak toxicity *in vivo*^21^, but it has been identified as the major toxic constituent in *Coptidis Rhizoma* extract^16^. In addition to berberine, other *Coptidis Rhizoma* alkaloids constituents may contribute to the acute toxicity of the extract^16^. However, given that the other toxic *Coptidis Rhizoma* constituents are less toxic than berberine and are present at much lower levels in the aqueous extract^16^,^22^, the increased acute oral toxicity of the *Coptidis Rhizoma* extract in comparison with that of pure berberine was not likely caused by the toxicity of other identified toxic *Coptidis Rhizoma* constituents. Instead, the acute toxicity of berberine was probably potentiated by the other constituents of the *Coptidis Rhizoma* extract. In brief, similar to the bioactivities of other natural product extracts, the bioactivities of the *Coptidis Rhizoma* extract diminished after purification to its most active constituent, berberine; however, the underlying mechanism remains unclear.

Bioavailability and related pharmacokinetic properties are crucial for the *in vivo* effects of a drug. Therefore, we aimed to explore the mechanism underlying the difference in acute oral toxicity between pure berberine and berberine in the *Coptidis Rhizoma* extract, with a focus on pharmacokinetics, in order to better understand the loss of bioactivity of natural products after purification.

## Results and Discussion

### Acute toxicity of orally administered *Coptidis Rhizoma* extract and pure berberine

The aim of this study was to evaluate differences in the acute oral toxicity of *Coptidis Rhizoma* extract and pure berberine. All mice died within 24 h after receiving the oral *Coptidis Rhizoma* extract at a dose of 3 g/kg; however, all mice that received equal pure berberine (618 mg/kg) or *Coptidis Rhizoma* alkaloid extract (1259 mg/kg) did not show toxic reactions after administration (Fig. 2A). This result confirmed that the *Coptidis Rhizoma* extract was more toxic than pure berberine in mice. Given that berberine is the dominant toxic constituent of the *Coptidis Rhizoma* extract^16^, our results also suggest that the acute toxicity of berberine was potentiated by other constituents in the extract. Other alkaloids in the extract were excluded as potentiators of berberine toxicity, because the alkaloid extract of *Coptidis Rhizoma* did not show increased acute toxicity in comparison with that of pure berberine.

### Plasma exposure of berberine in mice

As shown in Fig. 2B, the plasma berberine exposure level of the mice that received 3 g/kg *Coptidis Rhizoma* extract via oral administration was much higher than that of the mice that received 618 mg/kg pure berberine (AUC_0–4_ _h_: 338.9 vs. 22.1 ng·h/mL, p < 0.01). These results indicated that the difference in the toxicity of the *Coptidis Rhizoma* extract and pure berberine was associated with pharmacokinetic differences.

**Figure 1:**
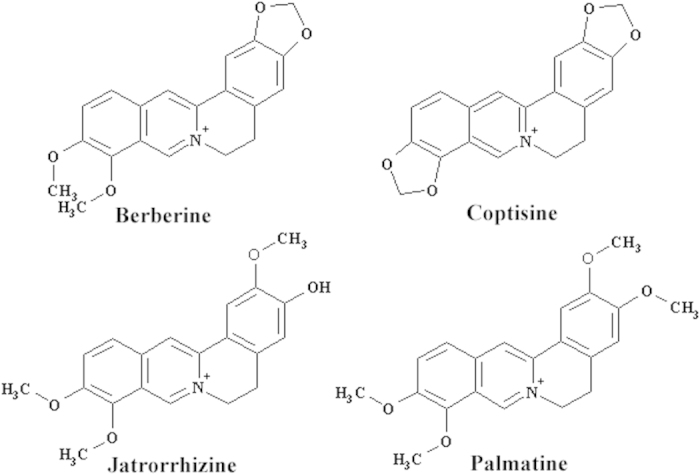
Structures of major alkaloids in *Coptidis Rhizoma*.

The possible causes of plasma berberine exposure difference include (1) conversion of other alkaloids in the extract to berberine in the body and (2) improvement of the pharmacokinetic properties of berberine by other materials in the extract. Given the structures of the other major compounds shown in Fig. 1, metabolic conversion of these compounds to berberine may be excluded. Therefore, the results described above suggest that the pharmacokinetic properties of berberine were improved by other constituents in the extract. Pharmacokinetic interactions could occur during the absorption, distribution, metabolism, and excretion of the compounds in the *Coptidis Rhizoma* extract. However, given that the difference in berberine plasma exposure levels produced by the *Coptidis Rhizoma* extract and pure berberine was mainly determined based on the absorption phases of the plasma exposure-time curves in this study, we therefore focused on the absorption process in subsequent studies.

### *In vitro* absorption and efflux of berberine

As shown in Fig. 3A,B, absorption of berberine from the *Coptidis Rhizoma* extract was significantly greater than that of pure berberine at both concentrations (both p < 0.01). To quantify the increase in berberine absorption, the ratio of berberine absorbed from the *Coptidis Rhizoma* extract to berberine absorbed after administration of pure berberine was calculated at the designated time points. After 75 min, the berberine absorption ratios for *Coptidis Rhizoma* extract to pure berberine at the low and high concentrations were 2.6 and 18.5, respectively. Based on these ratios, the improvement of berberine absorption in the higher concentration group was determined to be significantly better than that in the lower concentration group (p < 0.01, two-way ANOVA), indicating that the improvement in berberine absorption from the *Coptidis Rhizoma* extract was concentration-dependent.

Some compounds in the *Coptidis Rhizoma* extract showed inhibitory effects on P-gp, including 5′-methoxyhydnocarpin (5′-MHC), which is produced with berberine by several medicinal plants from the genus *Berberis*^23^. Therefore, the improvement of berberine absorption from the *Coptidis Rhizoma* extract might be caused by decreased efflux of the absorbed berberine, because berberine is a P-gp substrate^24^. However, efflux of berberine after administration of the *Coptidis Rhizoma* extract was also significantly greater than berberine efflux following administration of pure berberine (p < 0.01) (Fig. 3C). At 75 min, the ratio of berberine efflux following administration of *Coptidis Rhizoma* extract to that following administration of pure berberine was 2.3, which was roughly equal to the ratio (2.6) calculated based on the *in vitro* absorption experiment at the low concentration (p > 0.05). These results indicated that in comparison to the results for pure berberine, berberine absorption and efflux following administration of the *Coptidis Rhizoma* extract were higher, indicating that increased berberine exposure observed *in vivo* after administration of the extract was not due to decreased berberine efflux.

### Solubility of berberine

Poor solubility is a persistent challenge in drug discovery^25^. Good solubility is the basis of favourable absorption. Therefore, we tested whether the solubility of berberine was improved by the other constituents of the *Coptidis Rhizoma* extract. Not surprisingly, the solubility of berberine in the *Coptidis Rhizoma* extract was much higher (5.9 or 17 times higher at the low and high concentrations, respectively) than that of an equal amount of pure berberine (p < 0.01, Fig. 4).

These results indicated that improved solubility contributed to the increased *in vitro* absorption of berberine in the *Coptidis Rhizoma* extract in comparison with that of pure berberine. Given the extensive first-pass elimination of berberine^26^, we doubted the contribution of the improved solubility of berberine in the *Coptidis Rhizoma* extract to the markedly increased berberine systemic exposure. We assumed that the obtained supernatant in the solubility assay was probably not a true solution; therefore, SEM and LSCM experiments were performed to obtain visual images of the extract solution, whereas DLS experiments were performed to obtain quantitative data regarding the extract solution.

### SEM, LSCM, and DLS observation of the *Coptidis Rhizoma* extract solution

In practice, SEM has been widely used to observe the shape and size distribution of plant-derived nanoparticles^27^,^28^. The SEM experiment showed that irregularly shaped particles in the nano-scale to micrometre range existed in the *Coptidis Rhizoma* extract solution, while pure berberine formed large-scale crystals (Fig. 5A).

Given that berberine has native fluorescence^29^, we performed the following LSCM experiments that directly helped observe the original structure of the particles in the solution. The results confirmed that particles in the nano-scale to millimetre/submillimetre range existed in the extract solution (Fig. 5B). After centrifugation at 24,000 × *g* for 6 min, the size of the particles distributed from nano-scale to micrometre range (Fig. 5C). The nano-scale particles in the LSCM experiments looked bigger than those in the SEM experiment. The difference might be caused by the lower resolution of LSCM in comparison with that of SEM. Importantly, it was not the nanoparticles themselves, but the materials attached to the nanoparticles, which emitted green fluorescence (Fig. 5C), indicating that berberine had accumulated around the nanoparticles. It should be noted that only the nano-scale particles showed strong green fluorescence, which indicated abundant berberine accumulation.

It was not surprising to find the large size distribution of the particles in both experiments. It is a common phenomenon that both the size and shape of particles extracted from plants can be highly variable. For example, particles isolated from Harungana^27^, *Hypericum perforatum*^28^, black tea infusion^30^, and English ivy (*Hedera helix* L.)^31^, all show broad size distribution.

The DLS experiment showed that the average size of the particles was approximately 500 nm (Fig. 5D), which was larger than the size obtained from the SEM experiment. The difference in the results from the DLS and SEM experiments might have been caused by the irregular shape, high concentration, polydispersity, and surface properties of the particles, as well as aggregation of the particles during the DLS analysis^32^. Given that nanoparticles are defined as <100 nm in one dimension or <1000 nm aggregates and agglomerates^33^, the DLS analysis confirmed the existence of the nanoparticles in the *Coptidis Rhizoma* extract solution.

### LSCM observation of frozen sections of the intestine

An LSCM study was performed to determine whether the nanoparticles in the *Coptidis Rhizoma* extract solution were absorbed by the intestine. The intestinal villi, but not the cell bodies of the enterocytes, were autofluorescent (Fig. 6A). In the berberine-treated group, the cell bodies of the enterocytes showed equally distributed green fluorescence, which suggested that berberine was taken up by the cells and dispersed in the cell body. However, in the *Coptidis Rhizoma* extract treated group (Fig. 6C), several fluorescent particles appeared in the cell bodies of the enterocytes, which indicated the intestinal absorption of the nanoparticles.

Given that the absorbed nanoparticles could be delivered to intracellular compartments such as lysosomes and trapped within them, recycled to the extracellular milieu, or delivered to other cells^34^, these results did not confirm that the absorbed particles would enter the circulation and finally contribute to *in vivo* berberine exposure.

### Effects of high-speed centrifugation on LSCM observations, pharmacokinetics, and acute toxicity of the *Coptidis Rhizoma* extract solution

We attempted to remove the particles from the *Coptidis Rhizoma* extract solution by using high-speed centrifugation. However, although larger particles in the millimetre/submillimetre range were completely removed, nanoparticles were present even after centrifugation at 300,000 × *g* for 30 min (Fig. 7B). Plasma berberine exposure (Fig. 8A) and acute berberine toxicity (Fig. 8B) were not affected by centrifugation, which indicated that the particles in the millimetre/submillimetre range were not crucial for *in vivo* berberine exposure and the toxic effects of berberine. The relatively large size restricted the intestinal absorption of big particles^34^, while slight berberine loading (Fig. 5B) further limited their contribution to *in vivo* berberine exposure.

### Effects of dilution on LSCM observations, plasma berberine exposure, and the acute toxicity of the *Coptidis Rhizoma* extract solution

**Figure 2:**
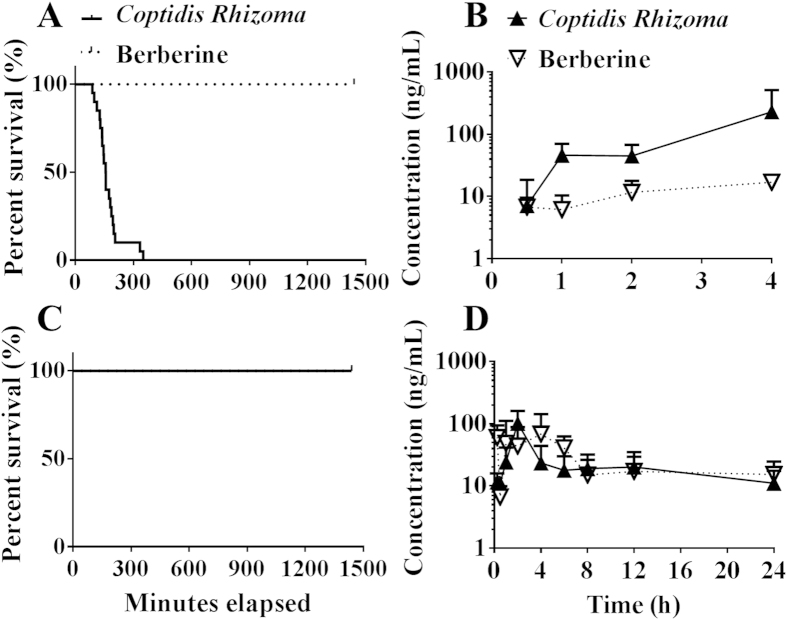
Acute toxicity and plasma exposure of berberine in mice that received the oral *Coptidis Rhizoma* extract or pure berberine (3 g/kg or 618 mg/kg, respectively, in A&B; 1 g/kg or 206 mg/kg, respectively, in C & D) (mean ± SD, n = 20 in A&C, n = 6 or 8 for each time point in B or D, respectively).

**Figure 3:**
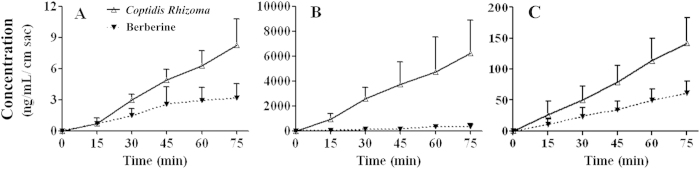
Comparative studies on the *in vitro* absorption (**A,B**) and *in vitro* efflux (**C**) of berberine in the *Coptidis Rhizoma* extract and pure berberine (mean ± SD, n = 6). (**A**) *in vitro* absorption of berberine in the extract (50 μg/mL) and pure berberine (10.3 μg/mL); (**B**) *in vitro* absorption of berberine in the extract (50 mg/mL) and pure berberine (10.3 mg/mL); (**C**) *in vitro* efflux of berberine in the extract (50 μg/mL) and pure berberine (10.3 μg/mL).

**Figure 4:**
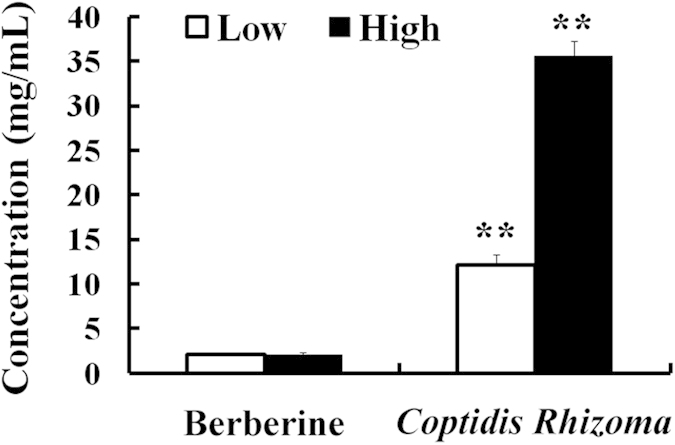
Aqueous solubility of berberine and berberine in *Coptidis Rhizoma* extract (mean ± SD, n = 3). Low: 50 mg of the *Coptidis Rhizoma* extract or 10.3 mg berberine was added to 1 mL of water; high: 150 mg of the *Coptidis Rhizoma* extract or 30.9 mg berberine was added to 1 mL of water. **p < 0.01 *vs.* Berberine.

**Figure 5:**
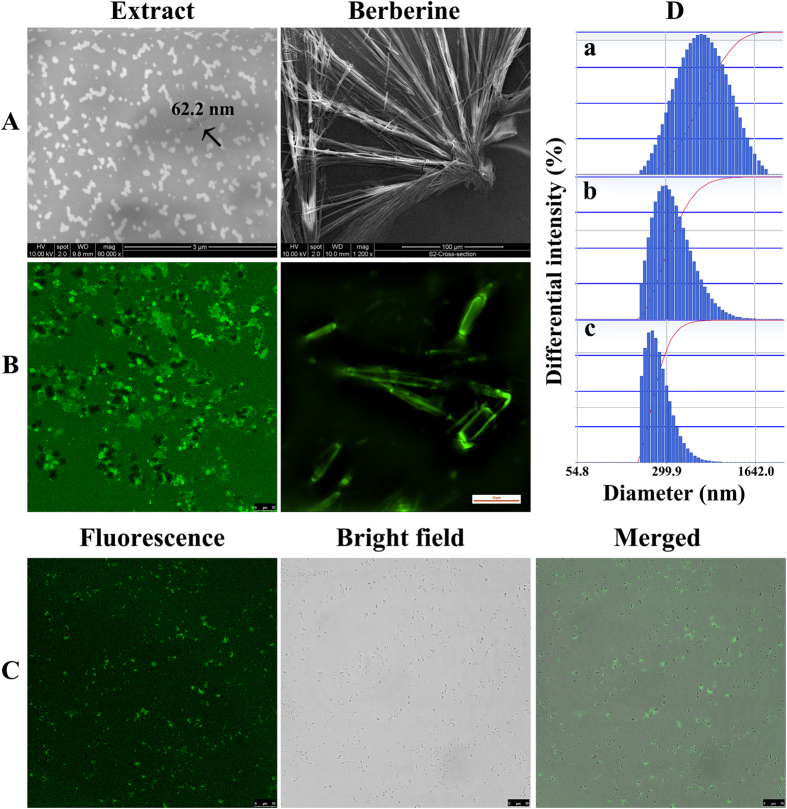
SEM (A), LSCM (**B,C**), and DLS (**D**) observation of the solutions. The solution in B was not centrifuged while the solutions in (**A,C,D**) were centrifuged at 24,000 × *g* for 6 min. Nanoparticles formed in the *Coptidis Rhizoma* extract (150 mg/mL), while berberine crystallized in water (30.9 mg/mL). The size of the particle indicated by the arrow was 62.2 nm. Data shown in D were acquired using a Delsa^TM^ Nano instrument (Beckman Coulter, USA). a: intensity distribution; b: volume distribution; c, number distribution.

**Figure 6:**
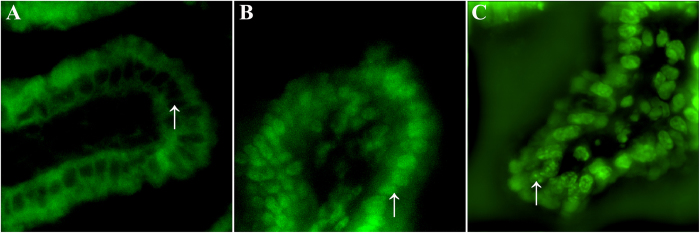
LSCM observation of the uptake of pure berberine (**B**) and nanoparticles in the *Coptidis Rhizoma* extract (**C**) by intestinal enterocytes. Mice were orally administered water (**A**), 618 mg/kg (30.9 mg/mL) berberine (**B**), or 3.0 g/kg (150 mg/mL) *Coptidis Rhizoma* extract solution (**C**). The mice were killed 1 h after administration of the test substance. The ileum from each mouse was cut into frozen sections that were observed using a confocal microscope system. Arrows indicate the cell bodies of the enterocytes.

**Figure 7:**
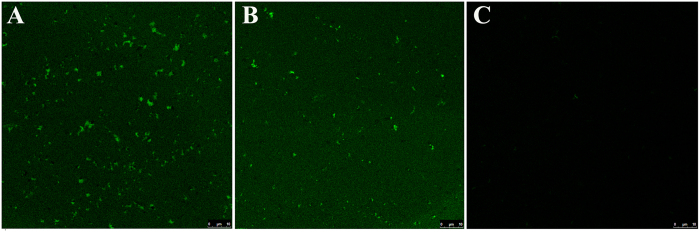
LSCM observation of the *Coptidis Rhizoma* extract solution. The extract was observed at a concentration of 150 mg/mL after centrifugation at 24,000 × *g* for 6 min (**A**) or centrifugation at 300,000 × *g* for 30 min (**B**); the extract was observed at a concentration of 50 mg/mL and after centrifugation at 24,000 × *g* for 6 min (**C**).

**Figure 8:**
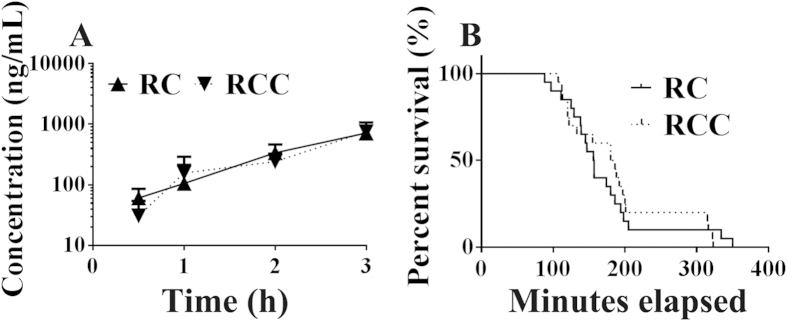
Plasma exposure of berberine (**A**) and acute toxicity (**B**) in mice that received the oral *Coptidis Rhizoma* extract (3 g/kg) with (RCC) or without (RC) centrifugation at 300,000 × g for 30 min (mean ± SD, n = 6).

When the *Coptidis Rhizoma* extract solution was diluted, the fluorescent particles in the solution nearly disappeared, indicating that the precipitates attached to the particles had been removed (Fig. 7C). All mice that received pure berberine or the *Coptidis Rhizoma* extract survived (Fig. 2C); furthermore, the difference in plasma berberine exposure following administration of the *Coptidis Rhizoma* extract and pure berberine disappeared (Fig. 2D, AUC_0–24_ _h_: 648.7 vs. 722.0 ng·h/mL, p > 0.05).

These results indicated that the action of the nanoparticles in this study was concentration-dependent. The nanoparticles only showed their enhancing effects at the highest tested concentration of the extract, in which the concentration of berberine was much higher than its saturated solubility. We guessed that the nanoparticles supplied a core for the precipitated berberine, which formed fine crystals. Interestingly, the process of producing the extract was similar to the “bottom up” approaches that have been developed to produce drug nanocrystals and nanosuspensions^35^. The concentration-dependent effects of the nanoparticles could also explain why *in vivo* berberine exposure in mice increased nonlinearly as the oral dose of the extract was increased and why the aqueous extract of *Coptidis Rhizoma* showed severe dose-dependent hepatic and pulmonary sub-chronic toxicity in Sprague-Dawley rats^16^,^18^.

### Isolation and identification of the nanoparticles in the extract

We attempted to purify the nanoparticles and identify the chemical nature of these nanoparticles. We obtained some brown powder after the dialysis and freeze-drying experiments. Subsequent LC-MS/MS and BCA assays, respectively, revealed that berberine only accounted for minimal (0.48%) while plant-derived protein accounted for most of the powder (almost 100%). It was not surprising to find that the nanoparticles were proteinaceous. It was recognized that particles isolated from plants always contain proteins^36^. For example, proteinaceous nanoparticles had been isolated from the adventitious roots of English ivy (*Hedera helix* L.)^37^.

**Figure 9:**
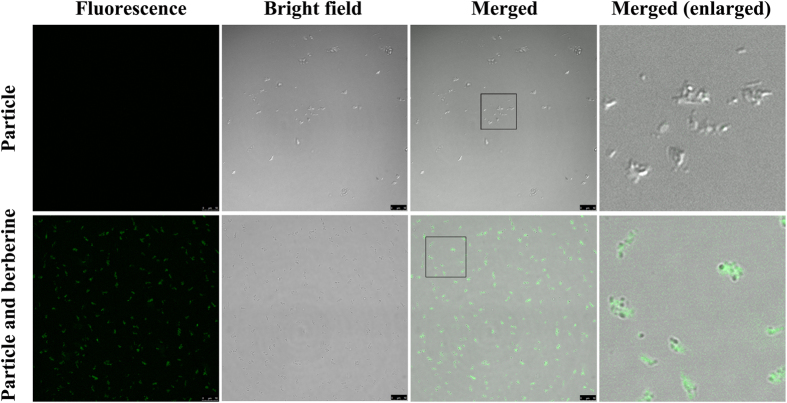
LSCM observation of the purified nanoparticles with (lower row) or without (upper row) berberine.

The LSCM experiment showed that the nanoparticles did not show any fluorescence, which indicated the absence of berberine (Fig. 9, upper row). However, the nanoparticles could capture berberine when they were dissolved together in water (Fig. 9, lower row).

The use of naturally occurring organic nanoparticles in medicine has drawn increasing interest in recent years. For example, spherical proteinaceous nanoparticles isolated from the adventitious roots of English ivy (*Hedera helix* L.) showed decreased cell toxicity and were easily degradable in comparison with metal nanoparticles^37^,^38^. Nanoparticles secreted from the carnivorous fungus *Arthrobotrys oligospora*, which has polysaccharides as its main chemical component, have been developed as bioactive nanocarriers^39^. Nanoparticles isolated from tea were able to form complexes with doxorubicin (DOX) and increased cellular DOX uptake by acting as nano-carriers^40^. Plant extracts have also been extensively used in “green synthesis” of nanoparticles as stabilizers/emulsifiers^41^,^42^. Nanocarrier-based oral drug delivery shows several advantages over other oral delivery methods, including enhanced absorption and bioavailability due to decreased metabolism and reduced drug efflux during intestinal absorption^43^.

## Conclusions

Proteinaceous nanoparticles were confirmed in the aqueous solution of the *Coptidis Rhizoma* extract by the SEM, LSCM, DLS, and BCA experiments. Furthermore, the following results suggest that the nanoparticles contributed to the increased absorption, plasma exposure, and acute oral toxicity of berberine in the *Coptidis Rhizoma* extract by acting as concentration-dependent carriers that facilitated berberine absorption: (1) the nanoparticles were proteinaceous and themselves did not emit green fluorescence like berberine did, which indicated that the nanoparticles were not composed of berberine itself; (2) the nanoparticles could absorb berberine; (3) the LSCM experiment showed that berberine was absorbed in the mouse intestine with the nanoparticles as a whole; and (4) the differences in acute oral toxicity and plasma exposure between the extract and pure berberine were only detected at high dosages.

In conclusion, our results clearly indicated that the proteinaceous nanoparticles in the *Coptidis Rhizoma* extract were crucial for the enhanced plasma exposure and biological activities of berberine, because they acted as natural drug carriers that increased berberine absorption. Our findings explain why the acute oral toxicity of the *Coptidis Rhizoma* extract decreased after purification. As mentioned above^8^,^9^,^10^,^11^,^12^,^13^,^14^, the pharmacokinetic properties of some pure active compounds in other natural products are also markedly different from that of crude extracts. We believe that the results of our studies will encourage more related studies.

## Materials and Methods

### Materials

*Coptidis Rhizoma* (*Coptis chinensis* Franch.) was purchased from Shanghai Kang Qiao Herbal Pieces Co., Ltd. (China), which is a GMP-certificated manufacturer. The herb was authenticated by Prof. Zhi-Li Zhao of the Department of Botany, Shanghai University of Traditional Chinese Medicine according to *The Pharmacopoeia of People’s Republic of China* (2010 edition). All *Coptidis Rhizoma* alkaloid standards were obtained from the National Institute for the Control of Pharmaceutical and Biological Products (Beijing, China). Carbamazepine and the QuantiPro bicinchoninic acid (BCA) assay kit were purchased from Sigma-Aldrich (St. Louis, MO, USA). Acetonitrile was purchased from Merck (Darmstadt, Germany). Tissue-Tek O.C.T. Compound was obtained from Sakura Finetek (Torrance, CA, USA). The pure water used in the current study was prepared using a Milli-Q system (Millipore Corporation, Billerica, MA, USA). All other materials were of analytical grade or higher.

### Standardized preparation and quality control of the *Coptidis Rhizoma* extracts^16^

The crude extract of *Coptidis Rhizoma* (designated *Coptidis Rhizoma* extract in this report) was prepared as follows: briefly, pieces of *Coptidis Rhizoma* were extracted twice with boiled alcohol (50% [v/v], diluted with water) for 1.5 h (for a total of 3 h). The extracted mixtures were filtered and dried under vacuum at 60 °C. Using high-performance liquid chromatography (HPLC) with ultraviolet detection, the contents of berberine, coptisine, palmatine, and jatrorrhizine in the extract powder were determined to be 20.62%, 3.51%, 2.64%, and 2.53%, respectively.

The alkaloid extract of *Coptidis Rhizoma* was prepared as follows: briefly, a tenfold mass of 0.5% sulphuric acid was added to the *Coptidis Rhizoma* extract and the mixture was centrifuged at 50 g for 15 min after being kept undisturbed for 1 h. The pH of the supernatant was adjusted to 7 with milk of lime, a slurry obtained by mixing dry hydrated lime and water. The resulting mixture was centrifuged at 1000 g for 15 min after being kept undisturbed for 1 h. The pH of the supernatant was adjusted to 1 with hydrochloric acid and sodium chloride was added to adjust the salinity of the solution up to 10%. After being kept undisturbed for 24 h, the mixture was filtered. The pH of the sediment was adjusted to 5 by washing with water. The sediment was dried under reduced pressure at 60 °C for approximately 7 h. The alkaloid extract yield was 8.5%. The contents of berberine, coptisine, palmatine, and jatrorrhizine in the alkaloid extract of *Coptidis Rhizoma* were 49.1%, 10.8%, 3.3%, and 4.3%, respectively.

The extracts and berberine used in all following studies consisted of the dried powder dissolved in water.

### Animals

Grade II mice (Kun-Ming [KM], 20–22 g) were purchased from Shanghai Slac Laboratory Animal Co., Ltd. (Shanghai, China). Male and female mice were used in this study. The mice were housed in an air-conditioned room at 22–24 °C under a 12-h dark/light cycle and given food and water *ad libitum*. The mice were fasted overnight before the experiments. All animal experimental protocols were approved by the Institutional Animal Care and Use Committee of Shanghai University of Traditional Chinese Medicine (Approval Number: 2015007), and all experiments were performed according to the guideline of this committee.

### Acute oral toxicity assay

The mice (n = 20 mice per group, 10 males and 10 females) were orally administered the test materials and the reactions of the mice were recorded for 7 days. Using this method, the acute oral toxicities of the following materials were tested at the dose volume of 0.2 mL/10 g body weight (dissolved in water) and compared: (1) the *Coptidis Rhizoma* extract (1 or 3 g/kg); (2) the supernatant of the *Coptidis Rhizoma* extract (3 g/kg) after centrifugation (300,000 *g*, 30 min, 4 °C); (3) pure berberine (206 or 618 mg/kg, equal to the dose of berberine in the 1 and 3 g/kg extracts, respectively); (4) alkaloid extract of *Coptidis Rhizoma* (1259 mg/kg, equal to the dose of berberine in the 3 g/kg extract).

### LC-MS/MS method^44^

A Shimadzu Prominence UFLCXR series HPLC (Shimadzu, Japan) and a Thermo Scientific TSQ Quantum Ultra mass spectrometer (Thermo Scientific, Waltham, MA, USA) equipped with an electrospray ionization (ESI) source were used. The samples were precipitated with 3 volumes of acetonitrile. Carbamazepine was used as the internal standard. After centrifugation (24,000 *g*, 6 min, 4 °C), the supernatant was mixed with an equal volume of water and 10-μL samples were injected into the LC-MS/MS system. The samples were eluted through a Hypersil Gold (C18) analytical column (5 μm, 100 × 2.1 mm) with a gradient of the aqueous phase (0.08% v/v formic acid and 2 mM ammonium acetate) and the acetonitrile phase (0 min, 85:15; 7 min, 32:68; 7.01 min, 85:15; 10 min, 85:15) at a flow rate of 0.3 mL/min. The ESI source was set to positive ion mode. Data acquisition was performed in the multiple reaction monitoring mode of the selective mass transition for each compound. The transitions from the precursor ions to the protonated fragment product ions were monitored: m/z 336.15 → m/z 322.28 for berberine and m/z 237.00 → m/z 194.31 for carbamazepine. The linear dynamic range for berberine in the tested biological samples was 0.5 to 500 ng/mL. The quality control samples were prepared at 3 different concentrations. The accuracy, precision, recovery, and stability tests all met the requirements of quantitative determination in biological samples.

### Pharmacokinetics in mice

Male and female mice were randomly divided into groups (n = 6 or 8 mice per group). The mice were orally administered the test materials: (1) the *Coptidis Rhizoma* extract solution (1 or 3 g/kg); (2) the *Coptidis Rhizoma* extract solution (3 g/kg) after centrifugation (300,000 × *g*, 30 min, 4 °C); or (3) pure berberine (206 or 618 mg/kg, equal to the berberine in the 1 or 3 g/kg *Coptidis Rhizoma* extract, respectively). Blood was collected into heparinized tubes at the designated time points, which were (1) 0.25, 0.5, 1, 2, 4, 8, 12, and 24 h for 1 g/kg *Coptidis Rhizoma* extract solution and 206 mg/kg pure berberine; (2) 0.5, 1, 2, and 4 h for 3 g/kg *Coptidis Rhizoma* extract solution and 618 mg/kg pure berberine; and (3) 0.5, 1, 2, and 3 h for 3 g/kg *Coptidis Rhizoma* extract solution with or without centrifugation. The last time point was selected to allow sampling prior to death induced by the high dose of the extract. After each sample was centrifuged (24,000 × *g*, 6 min, 4 °C), plasma was collected and stored at −80°C until the LC-MS/MS analysis.

### Berberine absorption across everted mouse gut sacs^44^

Mice were divided into 4 groups (n = 6 mice per group, 3 males and 3 females) that were used to study absorption of the berberine in the *Coptidis Rhizoma* extract (50 μg/mL or 50 mg/mL) and equal pure berberine (10.3 μg/mL or 10.3 mg/mL, respectively). The mice were sacrificed and the ileum was removed 5 cm above the caecum after laparotomy. The ileum was washed with chilled saline and everted. A 12-cm segment was cut and ligated at one end. The everted gut sac was filled on the serosal side (inside) with 1 mL of blank Krebs-Ringer buffer (containing 118 mM NaCl, 25 mM NaHCO_3_, 1.2 mM MgSO_4_, 2.5 mM CaCl_2_, 11 mM glucose, 1.2 mM KH_2_PO_4_, and 4.7 mM KCl, pH 6.8) and the other end was tightly ligated to create a gut sac. The sac was immediately incubated for 5 min at 37 °C in a Magnus bath containing 20 mL of oxygenated *Coptidis Rhizoma* extract (50 mg/mL or 50 μg/mL, dissolved in Krebs-Ringer buffer) or pure berberine. After incubation, aliquots of buffer (100 μL) were taken from the serosal side every 15 to 75 min and replaced with an equal volume of blank Krebs-Ringer buffer. The berberine concentration of each sample was measured using LC-MS/MS.

### Berberine efflux across everted mouse gut sacs^44^

The mice were divided into 2 groups (n = 6 mice per group, 3 males and 3 females). The intestines were collected as described above. The everted gut sac was filled on the serosal side (inside) with 1 mL of *Coptidis Rhizoma* extract (50 μg/mL) or equal pure berberine (10.3 μg/mL). The sac was immediately incubated at 37 °C in a Magnus bath containing 20 mL of oxygenated Krebs-Ringer buffer. All intestines were pre-incubated in Krebs-Ringer buffer for 5 min. After the incubation, aliquots (100 μL) were taken from the mucosal side (outside) every 15 to 75 min and replaced with the same volume of blank Krebs-Ringer buffer. The berberine concentration of each sample was measured using LC-MS/MS.

### Solubility of berberine^45^

To study the equilibrium solubility of berberine in the *Coptidis Rhizoma* extract, the *Coptidis Rhizoma* extract (50 mg or 150 mg) was added to 1 mL of water. To study the solubility of pure berberine, 10.3 or 30.9 mg berberine was added to 1 mL of water. The berberine concentrations were in accordance with those used in the pharmacokinetic and acute oral toxicity studies. The suspensions were subjected to ultrasound for 1 h followed by being kept undisturbed at room temperature (about 22 °C) for 5 h. The samples were centrifuged at 24,000 × *g* for 6 min and the supernatants were collected. After dilution, the berberine concentration of each sample was measured using LC-MS/MS. All experiments were performed in triplicate.

### Scanning electron microscopic (SEM) observation

The *Coptidis Rhizoma* extract (150 mg) or pure berberine (30.9 mg) was added to 1 mL of water. The concentrations were in accordance with those used in the pharmacokinetic and acute oral toxicity studies. The suspensions were subjected to ultrasound for 1 h followed by centrifugation at 24,000 × *g* for 6 min. Aliquots (10 μL) of the supernatants were mounted on double-stick carbon tape fastened to specimen holders and dried. The dried spots were examined and photographed under an FEI Quanta 400 F scanning electron microscope (FEI Co., Hillsboro, OR, USA) operating at 10 kV.

### LSCM observation of the extract and pure berberine solution

The *Coptidis Rhizoma* extract (150 mg) or pure berberine (30.9 mg) was added to 1 mL of water. The concentrations were in accordance with those used in the pharmacokinetic and acute oral toxicity studies. The suspensions were subjected to ultrasound for 1 h followed by centrifugation at 24,000 × *g* for 6 min. Aliquots (1.5 μL) of the supernatant were transferred to clean slides and carefully sealed using a coverslip and nail polish. The specimens were imaged using a Leica TCS SP8 confocal laser-scanning microscope (Leica, Wetzlar, Germany). The excitation wavelength was fixed at 488 nm and the emission wavelength ranged from 500 to 545 nm^29^.

### LSCM observation of intestinal uptake of the extract and pure berberine

Mice were orally administered water, the *Coptidis Rhizoma* extract solution (3 g/kg), or the berberine solution (618 mg/kg). One hour later, they were killed and the ileums were removed 5 cm above the caecum after laparotomy. A 0.5-cm segment was cut from the ileum, immersed into embedding medium (the O.C.T. compound), and frozen in liquid nitrogen. Frozen intestine samples were cut into 10-μm sections that were placed onto polylysine-coated slides. The specimens were carefully sealed using a coverslip and nail polish. Then the specimens were observed using a Nikon N-SIM microscope (Nikon, Tokyo, Japan). The excitation wavelength was fixed at 488 nm and the emission wavelength was in the range of 500 to 545 nm^29^.

### Dynamic light scattering (DLS) experiment

The *Coptidis Rhizoma* extract (150 mg) or pure berberine (30.9 mg) was added to 1 mL of water. The concentrations were in accordance with those used in the pharmacokinetic and acute toxicity studies. The suspensions were subjected to ultrasound for 1 h followed by centrifugation at 24,000 × *g* for 6 min. The supernatants were collected for the DLS experiments, which were performed using a Delsa Nano C Particle Analyzer (Beckman Coulter, Brea, CA, USA). The size of the particles was calculated using the manufacturer’s software. For the analysis, a sufficient sample volume was used to completely cover the electrodes of the cell. To avoid air bubbles in the cell, the sample was injected slowly and analysis was only carried out if there were no visible air bubble inclusions. After successful inspection, the cell was placed into the analyzer and equilibrated at 20 °C (close to the average temperature in the laboratory) for 2 min prior to the particle size measurements. All experiments were performed in 5 replicates.

### Isolation and identification of the nanoparticle in the extract

The *Coptidis Rhizoma* extract was dissolved in ultrapure water at the concentration of 150 mg/mL. The suspensions were subjected to ultrasound for 1 h followed by centrifugation at 24,000 × g for 6 min. The supernatants were collected for the dialysis experiment, which was performed using a ready-to-use laboratory dialysis device (Float-A-Lyzer G2, Spectrum Labs, LA, USA) for 3 d at room temperature. The free small molecules, such as alkaloids, were removed by dialysis using the 300 KD molecular weight cut-off tubing against ultrapure water. After that, the entrapped solution was lyophilized at −40 °C to obtain some brown powder. Then, the content of berberine was measured using LC-MS/MS and the content of protein was measured using a QuantiPro^TM^ BCA assay kit with bovine serum albumin as the standard. Furthermore, the obtained powder was dissolved with or without pure berberine (10 mg/mL) in ultrapure water and observed using LSCM.

### Statistical analysis

The results were expressed as mean ± S.D. values. A Log-Rank analysis was performed to compare the survival distributions of the groups of mice. Student’s t-test was used to compare the means of the treatment and control groups. ANOVA and Dunnett’s test were applied to compare multiple means. Results of p < 0.05 were considered significant.

## Additional Information

**How to cite this article**: Ma, B.-L. *et al.* Naturally occurring proteinaceous nanoparticles in *Coptidis Rhizoma* extract act as concentration-dependent carriers that facilitate berberine absorption. *Sci. Rep.* **6**, 20110; doi: 10.1038/srep20110 (2016).
